# The Influence of Thyroactive Substances on the Induction of Cervico-vaginal Tumours in Intact and Castrate Rats

**DOI:** 10.1038/bjc.1970.62

**Published:** 1970-09

**Authors:** Cora P. Cherry, A. Glucksmann

## Abstract

The effect of the administration of L-thyroxine and of methylthiouracil alone, together, in combination with stilboestrol or in the perinatal period on the induction of cervico-vaginal tumours by weekly local applications of DMBA was investigated in intact and castrate rats and compared with carcinogenesis in animals not additionally treated.

In intact rats the rate of sarcoma induction is accelerated by methylthiouracil, delayed and reduced by methylthiouracil plus L-thyroxine and delayed by perinatal injection of either L-thyroxine or methylthiouracil. In castrates sarcoma induction is accelerated and increased by L-thyroxine, methylthiouracil and by combination of either substance with stilboestrol; it is accelerated but not significantly increased by combined treatment with the thyroactive compounds.

The incidence of epithelial neoplasms is accelerated and increased in intacts and in castrates by methylthiouracil. This effect is slightly reduced in intacts but potentiated in castrates by additional stilboestrol treatment as well as by administration of L-thyroxine plus methylthiouracil.

The incidence of sarcomas is significantly greater in intact than in spayed rats not additionally treated, greater in castrates than in intacts given L-thyroxine ± stilboestrol and not significantly different in intacts and castrates with any of the other additional medications. For epithelial tumours the incidence is low and similar in both groups without additional treatment, greater in spayed than intact animals given methylthiouracil plus stilboestrol or plus L-thyroxine.

The influence of the thyroactive compounds on the induction of epithelial and sarcomatous tumours is not correlated with their effect on gain in body weight nor on growth of the stroma and of the epithelium of the vagina, cervix and uterus. Changes induced in the thyroid gland and the hypophysis are not correlated with those on carcinogenesis.

Central and local factors may account for the differential response in carcinogenesis of intacts and castrates as well as of the epithelial and connective tissue of the cervico-vaginal tract to medication with thyroactive compounds.


### THE INFLUENCE OF THYROACTIVE SUBSTANCES ON THE INDUCTION OF CERVICO-VAGINAL TUMOURS IN INTACT AND CASTRATE RATS

CORA P. CHERRY* AND A. GLUCKSMANN†

*From the Strangeways Research Laboratory, Cambridge*

Received for publication May 6, 1970

**SUMMARY.—The effect of the administration of L-thyroxine and of methylthiouracil alone, together, in combination with stilboestrol or in the perinatal period on the induction of cervico-vaginal tumours by weekly local applications of DMBA was investigated in intact and castrate rats and compared with carcinogenesis in animals not additionally treated.**

**In intact rats the rate of sarcoma induction is accelerated by methylthiouracil, delayed and reduced by methylthiouracil plus L-thyroxine and delayed by perinatal injection of either L-thyroxine or methylthiouracil. In castrates sarcoma induction is accelerated and increased by L-thyroxine, methylthiouracil and by combination of either substance with stilboestrol; it is accelerated but not significantly increased by combined treatment with the thyroactive compounds.**

**The incidence of epithelial neoplasms is accelerated and increased in intacts and in castrates by methylthiouracil. This effect is slightly reduced in intacts but potentiated in castrates by additional stilboestrol treatment as well as by administration of L-thyroxine plus methylthiouracil.**

**The incidence of sarcomas is significantly greater in intact than in spayed rats not additionally treated, greater in castrates than in intacts given L-thyroxine $\pm$ stilboestrol and not significantly different in intacts and castrates with any of the other additional medications. For epithelial tumours the incidence is low and similar in both groups without additional treatment, greater in spayed than intact animals given methylthiouracil plus stilboestrol or plus L-thyroxine.**

**The influence of the thyroactive compounds on the induction of epithelial and sarcomatous tumours is not correlated with their effect on gain in body weight nor on growth of the stroma and of the epithelium of the vagina, cervix and uterus. Changes induced in the thyroid gland and the hypophysis are not correlated with those on carcinogenesis.**

**Central and local factors may account for the differential response in carcinogenesis of intacts and castrates as well as of the epithelial and connective tissue of the cervico-vaginal tract to medication with thyroactive compounds.**

THIS investigation was undertaken for two reasons:

(1) Previous experiments using oestrogens, testosterone, progesterone, cortisone have shown that the hormonal effects on carcinogenesis do not parallel those on the normal target organs. Metabolic rather than specific hormonal actions on

* Working with a grant from the British Empire Cancer Campaign for Research.
† Gibb Senior Fellow, British Empire Cancer Campaign for Research.

normal target organs may be responsible for the discrepancy in the influence of endocrines on normal tissues and carcinogenesis in them. Thus the goitrogen methylthiouracil which slows down growth of the body, may retard, while its antagonist L-thyroxine may promote carcinogenesis.

(2) There are contradictory reports that the thyroid in man and animals influences incidence and growth rate of carcinomas (Wilkins and Morton, 1963). The geographical distribution of cancer appears to be correlated with the incidence of goitres (Spencer, 1954); thyroid anomalies at post-mortem are 4 times more frequent in patients with cancer than in those dying from other diseases. Administration of L-thyroxine has been reported to inhibit the induction and growth of sarcoma 180 (Williams and Williams, 1965) and of DBA-induced sarcomas (Bather and Franks, 1952) in mice, while thyroidectomy or feeding a goitrogen has been found to decrease the incidence of breast tumours in mice and rats (Vazquez-Lopez, 1949; Dubnik, Morris and Dalton, 1950; Jull and Huggins, 1960; Helfenstein, Young and Currie, 1962; Newman and Moon, 1968). The action of the thyroid and of thyroxine administration has been attributed to restriction of food intake, to the restriction of anaerobic respiration and to inhibition of dedifferentiation of carcinogenic tissue. If L-thyroxine has a similar action on the induction of cervico-vaginal tumours, inhibition of carcinogenesis may result. There are, however, reports that thyroidectomy like administration of L-thyroxine have only a minimal effect on the induction and growth of breast cancers in rats and mice (Jull and Huggins, 1960; Wilkins and Morton, 1963) and that this action is mediated through the effect on body weight (Gruenstein, Meranze, Acuff and Shimkin, 1968).

Castration inhibits and retards the appearance of sarcomas in the cervix and vagina of rats painted once weekly with DMBA (Glucksmann and Cherry, 1958). Continuous treatment with oestradiol or with stilboestrol restores the female genital tract from the atrophic castrate to the normal status, but does not enhance the induction of sarcomas. In fact in intact animals oestrogens inhibit the induction of tumours (Glucksmann and Cherry, 1968). On the other hand, adrenalectomy, or the administration of cortisone, pelvic or whole body irradiation of spayed rats does not alter the atrophic status of the female genital tract, but accelerates and increases the induction of sarcomas even beyond the rate obtained in intact rats treated with the carcinogen only (Cherry and Glucksmann, 1960). Intermittent administration of stilboestrol in doses insufficient to alter the castrate status of the vagina, cervix and uterus greatly accelerates and increases the induction of cervico-vaginal tumours and so does cholesterol. Thus stimulation of growth in the normal female genital tract by various hormones and other substances does not lead to increased carcinogenesis, nor is enhanced carcinogenesis paralleled by growth of the normal target organs.

On the other hand, castration causes atrophy of the epithelium and stroma of the cervico-vaginal tract and reduces the response to carcinogenic stimulation. Furthermore sarcomas predominate in intact and castrate rats treated with DMBA (9,10-dimethyl-1,2-benzanthracene) only. Some additional medications such as cholesterol, testosterone or intermittent administration of stilboestrol to castrates and of testosterone or progesterone to intact rats increase the incidence of epithelial tumours. In castrates testosterone and intermittent stilboestrol also promote the appearance of sarcomas while in intact animals testosterone at least delays their appearance. Thus the action of the various hormones on carcinogenesis is not correlated with their specific effects on the female genital tract, though there is an obvious connection in the case of castration. The connection is also evidenced by the fact that vulval tumours in intact and castrate rats treated by these additional means show only a slight response compared with those in the cervico-vaginal tract.

To find out whether metabolic effects of hormones might explain the differential action on normal tissues and those subjected to carcinogenic stimulation, modifications of metabolism, of immune responses and of central regulatory mechanisms by the pituitary and hypothalamus have been and are being investigated. The present report is concerned with the action of the goitrogen methylthiouracil and of L-thyroxine administered to adult intact and castrate rats (a) alone, (b) together, or (c) in combination with stilboestrol at the same time as DMBA, and (d) before DMBA in the perinatal period. Initially we expected that methylthiouracil might slow down or inhibit tumour induction in intact rats and that L-thyroxine might promote it in castrates. The experiments revealed, however, that methylthiouracil in spite of its inhibitory action on growth promotes the induction of sarcomas and of epithelial tumours in spayed as well as in intact animals, while L-thyroxine has only a minimal effect on tumour induction in intacts, but greatly stimulates the incidence of sarcomas in castrates.

### MATERIALS AND METHODS

Hooded rats of the Lister strain, random bred in this laboratory as a closed colony since 1940, were used for the experiments which extended over a period from 1955 to 1967. The rats were housed 7 to a cage and given water and food pellets of MRC-diet 86 *ad libitum*. In most of the experiments the thyroactive substances or stilboestrol were dissolved and administered in the drinking water. Only animals surviving for at least 100 days were considered at risk and the number of rats in the various treatment groups are given in Table I.

TABLE I.—*Treatment Groups, Number of Rats at Risk, Duration of Experiment, Average Diameter of Uterine Horns, and Incidence of Cervico-vaginal Sarcomas*

| Treatment groups (additional treatment) | | No. at risk | Duration of experiment (days) | Diameter of uterine horns | Sarcomas % ± S.E. |
|---|---|---|---|---|---|
| None | ♀ | 43 | 382 | 1·20* | 72± 6·85 |
| | ⚲ | 36 | 406 | 0·33 | 25± 7·20 |
| L-thyroxine | ♀ | 43 | 388 | 1·20 | 65± 7·28 |
| | ⚲ | 61 | 322 | 0·32 | 92± 3·48 |
| Methylthiouracil | ♀ | 42 | 328 | 1·00 | 77± 6·50 |
| | ⚲ | 40 | 323 | 0·29 | 90± 4·75 |
| Both | ♀ | 21 | 336 | 1·01 | 29± 9·90 |
| | ⚲ | 21 | 304 | 0·28 | 48±10·90 |
| L-thyroxine perinatally | ♀ | 25 | 328 | 1·03 | 36± 9·60 |
| Methylthiouracil perinatally | ♀ | 21 | 322 | 1·12 | 38±10·60 |
| Stilboestrol | ♀ | 21 | 329 | 1·00 | 14± 7·60 |
| | ⚲ | 38 | 396 | 1·01 | 26± 7·10 |
| Stilboestrol + L-thyroxine | ♀ | 20 | 373 | 1·20 | 55±11·13 |
| | ⚲ | 19 | 300 | 0·79 | 84± 8·41 |
| Stilboestrol + Methylthiouracil | ♀ | 21 | 325 | 1·00 | 62±10·60 |
| | ⚲ | 19 | 370 | 0·72 | 74±10·07 |
| Oestradiol perinatally | ♀ | 25 | 570 | 0·98 | 32± 9·30 |

* A diameter of 1·0 = 1·5 mm. in the histological specimen

Bilateral ovariectomy was performed with a dorsal approach under ether anaesthesia on rats aged 6 to 8 weeks. Carcinogenic treatment with a 1% solution in acetone of 9,10-dimethyl-1,2-benzanthracene (DMBA, Koch Light Ltd.) was started when intact and castrate animals were 2–3 months old or at the age of about 4 months when thyroactive substances were given for 70 days before starting intravaginal painting. The vagina was stretched open by dorsal flexion of the tail; the solution was applied by means of a cotton wool swab mounted on a thin wire rod and distributed through a rotary motion over the cervix, vagina and introitus. This procedure was repeated at weekly intervals for the life span of the animals.

*L-thyroxine sodium* B.P. (Eltroxin, Glaxo) was added to the drinking water (1 mg./1000 ml.) giving a daily dose per rat of approximately 20 $\mu$g. One group each of intact and castrate animals were treated additionally with 25 $\mu$g. of the hormone once weekly by intramuscular injection.

*Methylthiouracil* (B.D.H. 1 g./1000 ml.) was administered in the drinking water in a daily dose of about 20 mg. per rat.

*Stilboestrol* B.P. was given in the drinking water in a concentration of 0·1 mg./1000 ml. thus dosing each rat with about 2 $\mu$g. per day.

*Combined treatments.*—When two of the above substances were administered simultaneously in the drinking water the concentration of each solution was adjusted to arrive at the same dose as when each compound was given separately.

*Perinatal treatments.*—Rats were injected subcutaneously within 24 hours of birth either with 1 $\mu$g. of L-thyroxine sodium or 500 $\mu$g. of methylthiouracil and the same dose was repeated after 24 hours.

Some groups of intact and castrate females treated with thyroid hormone or the goitrogen were marked and weighed individually at weekly or fortnightly intervals to determine the effect on body growth.

All rats were examined at weekly intervals and sick animals or those with clinical signs of vulval or vaginal tumours were killed and a post-mortem performed. In addition to the organs of the genital tract from ovary to vulva the following tissues were taken for histological examination: pituitary, thyroid, thymus, lungs, liver, spleen, kidneys, adrenals, intestine, mesenteric, lumbar and inguinal nodes. The material was fixed in Zenker-acetic or Bouin's fluid, dehydrated, embedded in paraffin wax and sectioned at 6 or 8 $\mu$ depending on the organ; the endocrine glands were sectioned serially. Sections were stained with haematoxylin-eosin, carmalum-orange G-aniline blue, Van Gieson, Southgate's mucicarmine or the periodic acid-Schiff technique (PAS) after diastase digestion.

### RESULTS

### *Effects of thyroactive compounds on normal tissues*

*Genital tract.*—The administration of thyroactive substances either alone or in combination to adult rats or separately in the perinatal period has no effect on the normal histology of the ovaries; ova, primordial, Graafian and atretic follicles as well as *corpora lutea* are present. Additional treatment with the dose of stilboestrol used does not materially affect the histology nor does it cause ovarian abscesses.

Castration causes atrophy of the vaginal epithelium and reduces the width of the stroma of the uterus and of the vagina to 40% and 48% respectively of that in intact animals (Glucksmann and Cherry, 1958). The volume of all these target tissues is restored to intact proportions by additional weekly treatment of castrates with 3 $\mu$g of oestradiol monobenzoate i.m. or 14 $\mu$g. of stilboestrol per os. In the present experiments uterine measurements show that the thyroactive substances alone, in combination or perinatally have no such effect and the uterus remains atrophic (Table I). On the other hand, in castrates methylthiouracil as well as thyroxine inhibit to some extent the stilboestrol effect on the uterus (Table I).

Squamous metaplasia of the endometrial epithelium and the glands is not induced by the administration of thyroactive compounds. The changes in the vulval skin are identical with those described below.

*Skin.*—Methylthiouracil causes loss of hair and after prolonged administration a fairly generalized alopecia. Intact and castrate females appear to be affected equally even though castration like methylthiouracil causes aplasia or hypoplasia of the hair follicles. The skin in such animals assumes a yellowish colour as a result of carotinaemia. A single topical application of L-thyroxine to a bald area of skin in goitrous rats induces temporary regrowth of hair for at least two hair cycles. Concomitant methylthiouracil and thyroxine administration prevents loss of hair and the yellow pigmentation but combined treatment with stilboestrol and the goitrogen does not. No abnormal skin and hair changes occur in rats treated perinatally with methylthiouracil.

*Thyroid gland.*—Methylthiouracil causes marked enlargement of both lobes and of the isthmus of the thyroid; goitres appear in all intact and castrate rats thus treated for 114 to 388 days. The lobes are not always equally enlarged and the size of the goitres varies not necessarily with the duration of the treatment period. Histologically the predominant change is diffuse hyperplasia of the follicles and hypertrophy of the follicular cells with depletion of colloid giving a solid columnar pattern. In some instances cystic or papillary cystic nodules containing colloid secretion occur within the goitrous gland. The thyroid tumours invade the capsule of the gland and surrounding fat quite frequently and sometimes metastasize to the lungs. Additional treatment with stilboestrol does not prevent the formation of goitres in either intact or castrate animals (Table II). Combined methylthiouracil and thyroxine administration reduces the incidence of goitres but colloid secretion is often irregular, the gland is slightly enlarged and some goitres occur after about 40 weeks.

L-thyroxine sodium does not appear to alter the histological structure of the gland nor to affect the secretion of colloid. In both intact and castrate animals the gland shows larger peripheral and smaller central follicles which is typical for the rat's thyroid. Additional treatment with stilboestrol slightly inhibits secretory activity especially in intacts and this may be correlated with changes in the pituitary to be described below.

The thyroid gland of adult rats shows similar changes when either of the thyroactive substances are injected perinatally. Histologically the gland has a solid and compact appearance with reduced secretory activity although goitres do not occur even with methylthiouracil treatment.

Microscopical solid follicular adenomas have been found in the thyroids of a few animals in most experimental groups. Similar adenomas occur in control intact and castrate rats of our colony (Glucksmann and Cherry, 1968) and none of the treatments in the present series of experiments has altered their incidence. They are included as tumours of the thyroid in Table II.

TABLE II.—*Incidence of Thyroid and Pituitary Tumours and of Leukaemia*

| Treatment groups (additional treatments) | | Tumours of: Thyroid % | Pituitary % | Leukaemia % |
|---|---|---|---|---|
| None | ♀ | 2 | 0 | 2 |
| | ♀̸ | 3 | 0 | 11 |
| L-thyroxine | ♀ | 5 | 12 | 2 |
| | ♀̸ | 0 | 2 | 3 |
| Methylthiouracil | ♀ | 100 | 2 | 2 |
| | ♀̸ | 100 | 2 | 0 |
| Both | ♀ | 29 | 5 | 19 |
| | ♀̸ | 14 | 0 | 10 |
| L-thyroxine perinatally | ♀ | 12 | 4 | 12 |
| Methylthiouracil perinatally | ♀ | 33 | 10 | 14 |
| Stilboestrol | ♀ | 10 | 0 | 0 |
| | ♀̸ | 0 | 16 | 0 |
| Stilboestrol + L-thyroxine | ♀ | 0 | 5 | 5 |
| | ♀̸ | 5 | 0 | 0 |
| Stilboestrol + Methylthiouracil | ♀ | 100 | 5 | 0 |
| | ♀̸ | 100 | 5 | 0 |
| Oestradiol perinatally | ♀ | 16 | 8 | 0 |

*Pituitary*.—The anterior lobe enlarges after castration and contains a very large number of hypertrophied gonadotrophs as well as of castration cells. The thyroactive substances have no effect on the gonadotrophs and additional stilboestrol treatment fails to prevent enlargement of the gonadotrophs or the appearance of castration cells. Enlargement of the pituitary occurs also after methylthiouracil administration and this is accompanied in both intacts and castrates by the appearance of numerous thyroidectomy cells with very prominent and coarse PAS-positive granules. In both intact and castrate rats given L-thyroxine alone thyrotrophs are found although they are probably less prominent with smaller and more faintly stained granules. In intact females treated concomitantly with stilboestrol and L-thyroxine the thyrotrophs are more prominent than normal and often contain rather coarse PAS-positive granules not unlike the thyroidectomy cells. Their appearance may be related to the inhibition of secretion in the thyroid mentioned above.

Combined thyroxine and methylthiouracil administration to intact or castrate rats does not prevent the appearance of thyroidectomy cells although they are less numerous than with methylthiouracil alone. These pituitary changes may be correlated with the irregular colloid secretion in the thyroids and the induction of some goitres. Perinatal treatment with either thyroxine or the goitrogen has no obvious effect on gonadotrophs or thyrotrophs.

Adenomas have been observed in the pituitary of some rats in most of the experimental groups but, as with the thyroid, their incidence is similar to that in our intact and castrate control series (Table II). Some adenomas are very vascular, and replace most of the gland; others are solid, circumscribed and show variation in cell and nuclear size and mitotic activity. Some of their cells can be identified as either gonadotrophs or thyrotrophs by their PAS-positive granulation.

*Adrenals*.—In rats treated with methylthiouracil alone the glands have an abnormal dark brown colour. Solid adenomas occur in the cortex of some rats but their incidence is not correlated with any particular form of treatment nor does it differ from that in our control series.

*Breast tumours* do not occur in spayed females of our colony and none have been induced by any of the additional treatments. In intact controls the incidence of breast tumours has varied from 7% in 1955 to 10% in 1964. In the present series fibroadenomas of the breast have been found in 3 intact rats given L-thyroxine.

*Leukaemia.*—The incidence of leukaemia in the present experiments has varied from 0 to 19% (Table II) and falls within the control range as does the time when the disease becomes manifest. There is no evidence that DMBA application nor additional treatment with thyroactive substances and stilboestrol increases the incidence of leukaemias.

*Effects of hormonal treatments on gain in body weight*

Since some of the action of thyroactive substances on carcinogenesis have been attributed to their effect on body weight (Jull and Huggins, 1960; Gruenstein *et al.*, 1968), the weights of rats given L-thyroxine or methylthiouracil have been recorded for the duration of the experiment. In Fig. 1 the average weights for 4 groups of 21 animals each are contrasted. The initial weight and age of the rats varied to some extent and the growth curve in young animals is very steep as for instance in intacts given L-thyroxine. Rats on methylthiouracil remain small while those on L-thyroxine continue to grow. The gain in body weight is not correlated with the incidence of induced tumours, since these are of the same order in intacts and in castrates treated with thyroactive compounds (Fig. 2 and 8; Table III).

If the differences in initial weight are corrected by calculating the percentage increase in weight from an average of 190 $\pm$ 10 g. for a subsequent period of 28 weeks, the total gain in L-thyroxine treated rats is around 40% and in females with goitres only 14%. Additional data on the relation of body weight to induction of tumours obtained in as yet unpublished experiments are given in Table III, and show that tumour induction does not vary with percentage gain in weight.

FIG. 1.—Average weight of intact and castrate rats given L-thyroxine (L) or methylthiouracil (M).

TABLE III.—*Average Gain in Body Weight up to* 190 ± 10 *g. and for* 28 *Weeks after Reaching this Level*

| Additional treatment | | Initial weight | (a) weeks/% | (b) % | Sarcomas % ± S.E. | Papillomas + carcinomas % ± S.E. |
|---|---|---|---|---|---|---|
| L-thyroxine | ♀ | 124 | 4/13 | 35 | 65± 7·28 | 0 |
| | ♀̸ | 168 | 2/6·5 | 45 | 92± 3·48 | 16± 4·7 |
| Methylthiouracil | ♀ | 180 | — | 14 | 77± 6·50 | 45± 7·7 |
| | ♀̸ | 189 | — | 14 | 90± 4·75 | 30± 7·2 |
| Insulin | ♀ | 197 | — | 24 | 52±10·4 | 9± 6·0 |
| | ♀̸ | 117 | 4/15 | 45 | 85± 8·0 | 50±11·2 |
| Alloxan | ♀ | 124 | 16/3·3 | 20 | 65± 9·4 | 19± 7·7 |
| (sugar in urine) | ♀̸ | 156 | 4/5·5 | 43 | 95± 4·9 | 75± 9·7 |
| (urine negative | ♀ | 161 | 4/4·5 | 28 | 46±12·9 | 7± 6·6 |
| for sugar) | ♀̸ | 197 | — | 47 | 94± 5·9 | 25±10·8 |
| Growth hormone | ♀ | 189 | — | 48 | 43±10·8 | 28± 9·8 |
| | ♀̸ | 137 | 2/18 | 78 | 55±11·1 | 40±11·0 |

(a) Number of weeks and percentage gain per week to reach an average weight of 190 ± 10 g.
(b) Percentage gain in 28 weeks after reaching an average weight of 190 ± 10 g.

Thus the animals receiving growth hormone* grow fastest but have a lower incidence of sarcomas and of epithelial tumours than the methylthiouracil treated animals at the other end of the growth scale. The highest incidence of all tumours occurs in diabetic castrates whose increase in body weight is very much like that of castrates given L-thyroxine.

The initial gain in weight, *i.e.* until an average of 190 ± 10 g. is reached, appears to vary with the additional treatment rather than the level of the initial weight: in diabetic intacts of 124 g. it is only 3·3% while in L-thyroxine treated intacts of the same initial weight it is 13% per week; in insulin treated castrates of 117 g. it is 15% compared with 18% in castrates of 137 g. treated with growth hormone.

From the 190 g. level castrates gain twice as much as intacts receiving the same treatment, except that there is no difference in weight gain between intacts and castrates given the goitrogen and the difference is smaller in the thyroxine treated animals. Greater deposition of fat in castrates accounts to some extent for the difference in weight. With the exception of epithelial tumours and the weight increase in methylthiouracil medicated rats, the incidence of both sarcomas and epithelial tumours, and the gain in body weight, is always greater in castrates than in intacts.

*Effects on carcinogenesis*

The histogenesis and histology of sarcomas and of epithelial tumours induced by DMBA in the cervico-vaginal tract has been described in some detail in a previous publication (Glucksmann and Cherry, 1970) and the same range of tumours has been found in the present experiments. The additional treatment with thyroactive substances has not modified the type of induced tumours nor the process of histogenesis, though it has affected the rate of carcinogenesis particularly in castrates. As pointed out previously (Glucksmann and Cherry,

* A preparation of bovine growth hormone was kindly presented by Dr. A. E. Wilhelmi of Emory University, Atlanta, Georgia, on behalf of the Endocrinology Study Section of the National Institutes of Health, Bethesda, U.S.A.

1968), experiments repeated at intervals of up to 10 years have yielded the same results as regards tumour induction.

*Cervico-vaginal sarcomas.*—The effects of concomitant DMBA treatment with either L-thyroxine or methylthiouracil or with both substances are illustrated in Fig. 2. In intact rats L-thyroxine has little effect on the rate of appearance of sarcomas, while methylthiouracil shortens the induction period for the first tumours, but does not affect the subsequent accumulation of neoplasms which parallels that of intact rats not additionally treated ($H_2O$). The combined treatment prolongs the interval before the first sarcomas arise, but does not affect the rate of tumour induction. Methylthiouracil like L-thyroxine shortens the induction period of sarcomas in castrates and greatly increases the rate at which the tumours appear. This effect is significant in comparison with castrate as well as intact rats treated by DMBA only. The combined treatment still promotes sarcoma formation in castrates, but not to the same extent as does either treatment alone.

FIG. 2.—The induction of cervico-vaginal sarcomas in intact and castrate rats given methylthiouracil, L-thyroxine or both after weekly applications of DMBA.

The analysis for the three separate experiments of treating the rats with L-thyroxine added to the drinking water alone, or supplemented by intramuscular injections or started 70 days before DMBA painting is given as age-specific induction rates in Fig. 3. There is no significant difference between the various forms of treatments in either intact or castrate rats.

Fig. 4 gives a similar analysis of experiments with methylthiouracil, showing again little difference between treatments started 70 days before and at the same time as DMBA painting. The slight accelerating effect in intacts and the marked

FIG. 3.—Age-specific induction rates of cervico-vaginal sarcomas by weekly applications of DMBA in intact and castrate rats given L-thyroxine (L), *i.e.* the percentage of rats with tumours during consecutive 100 day periods plotted at the 50 day interval.

FIG. 4.—Age-specific induction rates of cervico-vaginal sarcomas by weekly applications of DMBA in intact and castrate rats given methylthiouracil (M).

acceleration and promotion of tumour induction in castrates are clearly demonstrated. The age-specific induction rates for additionally treated intacts are very close to those for castrates, while for L-thyroxine the intacts lag behind the castrates (Fig. 3).

The differences between intacts and castrates without additional treatment are significant at the 95% confidence level and the same holds true for L-thyroxine though this time in favour of castrates. The total incidence of sarcomas (Table I) is of the same order in intact and castrate rats treated with the goitrogen. Compared with animals not additionally treated the combined treatment decreases and slows down tumour formation in intacts but accelerates and slightly increases it in castrates. The final difference is not significant at the 95% confidence level but the duration of experiments with castrates not additionally treated exceeds by 100 days that with both thyroactive substances. Compared with the effect of either methylthiouracil or L-thyroxine alone, the combined treatment produces significantly fewer sarcomas in intacts and castrates for the same period of observation. The combination of thyroactive substances does not cancel out the separate effects, since there is a significant depression of sarcoma induction in intacts and a real acceleration in castrates (Fig. 2).

To test whether the promotion of growth of the tissues of the cervico-vaginal tract by oestrogens affects the induction of tumours by DMBA, stilboestrol was administered continuously to intact and castrate animals also given a thyroactive compound. Additional stilboestrol alone retards and decreases the incidence of sarcomas in intacts but has no effect in castrates (Fig. 5). Thus the difference in sarcoma induction between intacts and castrates disappears after oestrogenic treatment because of the depressing effect in intacts.

Combination of stilboestrol with L-thyroxine causes a very significant increase and acceleration of sarcoma induction in intacts compared with those given stilboestrol only, but a slight retardation and decrease compared with rats not given any additional treatment (Fig. 5). In castrates the combination greatly

FIG. 5.—The induction of cervico-vaginal sarcomas by weekly applications of DMBA in intact and castrate rats given L-thyroxine (L) plus stilboestrol (S).

increases the incidence and accelerates the appearance of sarcomas as compared with those given only stilboestrol or no additional treatment. The effect is about equal to that of L-thyroxine alone.

In similar experiments with methylthiouracil (Fig. 6) combination with stilboestrol greatly increases the incidence of sarcomas in intacts compared with stilboestrol alone but retards it in comparison with methylthiouracil. A similar retardation occurs in castrates though there is still a significant increase and acceleration in comparison with rats given stilboestrol or no additional medication.

FIG. 6.—The induction of cervico-vaginal sarcomas by weekly applications of DMBA in intact and castrate rats given methylthiouracil (M) plus stilboestrol (S).

The injection of oestradiol in the perinatal period greatly delays and decreases the incidence of sarcomas following painting with DMBA (Cherry and Glucksmann, 1968). To see whether treatment with thyroactive compounds in the perinatal period modifies the response to carcinogens because of changes in the pituitary and hypothalamus, experiments with these substances were performed. The injection of L-triiodothyronine in the perinatal period has been reported to affect the growth of rats, of their thyroid and the formation of acidophiles in the pituitary (Eayrs and Holmes, 1964). The results for treatment with methylthiouracil and L-thyroxine on sarcoma induction are the same (Fig. 7): compared with intact rats the rate is slowed down though not to the same extent as in oestrogenized animals. In all three instances the level of sarcoma incidence is close to that of castrates not additionally treated.

To sum up: compared with intact rats without additional treatment the rate of sarcoma induction is accelerated by the administration of methylthiouracil, delayed by the injection of methylthiouracil or L-thyroxine in the perinatal period

FIG. 7.—The induction of cervico-vaginal sarcomas by weekly applications of DMBA in rats treated perinatally with methylthiouracil (M) or L-thyroxine (L).

and delayed and significantly reduced by stilboestrol and by the administration of methylthiouracil plus L-thyroxine. Compared with castrates without additional treatment the induction of sarcomas is accelerated and increased by L-thyroxine, methylthiouracil and by either substance combined with stilboestrol; it is accelerated by combined treatment with methylthiouracil plus L-thyroxine and not changed by stilboestrol alone. The incidence of sarcomas is significantly greater in intacts than in castrates without additional treatment, greater in castrates than in intacts treated with L-thyroxine $\pm$ stilboestrol and not significantly different in intacts and castrates after administration of methylthiouracil $\pm$ stilboestrol, methylthiouracil plus L-thyroxine and by stilboestrol alone.

*Cervico-vaginal carcinomas and papillomas.*—As regards the promotion or inhibition by thyroactive compounds, carcinomas and papillomas can be considered together because on the whole the frequency of carcinomas increases with that of papillomas and follows it. The proportion of papillomas to carcinomas in the various experiments is given in Table IV. Without additional treatment the incidence of epithelial tumours is the same in intacts and castrates and amounts to about 10% (Fig. 8). Methylthiouracil accelerates and increases the incidence of epithelial neoplasms in castrates and intacts, while L-thyroxine does not materially alter the yield in either though there is a slight acceleration in tumour formation in castrates and an inhibition in intacts. Combined treatment with the thyroactive substances in spayed females potentiates the promoting effect of either alone with statistically significant differences in yield from either separately. In intact animals the effect of combined treatment is intermediate between that of either substance alone, and not significantly different from that of animals without additional medication.

As regards epithelial tumours methylthiouracil combined with stilboestrol potentiates the action of DMBA in spayed rats, but slightly reduces the methylthiouracil effect in intact animals (Fig. 9). Stilboestrol added to L-thyroxine

TABLE IV.—*Percentage of Rats with Cervico-vaginal Papillomas and Carcinomas*

| Additional treatment | | Papillomas % | Carcinomas % | Papillomas + carcinomas % ± S.E. |
|---|---|---|---|---|
| None | ♀ | 5 | 2 | 7± 3·9 |
| | ♀̸ | 11 | 0 | 11± 5·2 |
| L-thyroxine | ♀ | 0 | 0 | 0 |
| | ♀̸ | 14 | 2 | 16± 4·7 |
| Methylthiouracil | ♀ | 43 | 2 | 45± 7·7 |
| | ♀̸ | 25 | 5 | 30± 7·2 |
| Both | ♀ | 14 | 0 | 14± 7·6 |
| | ♀̸ | 33 | 24 | 57±10·8 |
| L-thyroxine perinatally | ♀ | 24 | 0 | 24± 8·6 |
| Methylthiouracil perinatally | ♀ | 5 | 0 | 5± 4·8 |
| Stilboestrol | ♀ | 5 | 0 | 5± 4·8 |
| | ♀̸ | 0 | 0 | 0 |
| Stilboestrol + L-thyroxine | ♀ | 10 | 0 | 10± 6·7 |
| | ♀̸ | 21 | 0 | 21± 9·3 |
| Stilboestrol + Methylthiouracil | ♀ | 19 | 10 | 29± 9·9 |
| | ♀̸ | 42 | 11 | 53±11·5 |
| Oestradiol perinatally | ♀ | 28 | 20 | 48±10·0 |

FIG 8.—The induction of cervico-vaginal carcinomas and papillomas in intact and castrate rats given methylthiouracil, L-thyroxine or both after weekly applications of DMBA.

slightly, but not significantly, promotes carcinogenesis in both groups of rats. Perinatal injection of either of the thyroactive compounds does not influence the subsequent induction by DMBA of epithelial neoplasms (Table IV).

If the action of additional medication with thyroactive compounds whether given alone, in combination or in addition to stilboestrol is compared in intact and in spayed rats, some interesting and significant differences are brought to light. Significantly more epithelial tumours are induced in the 160 castrates, *i.e.* 30% ± 3·6, than in the 147 intacts, *i.e.* 20% ± 3·3. The incidence of papillomas is not significantly different in the two groups (24% ± 3·4 in castrates and 18% ± 3·2 in intacts), nor is that of carcinomas although it rises by a factor of 3 from 2% ± 1·2 in intacts to 6% ± 1·9 in castrates. In a similar calculation for sarcomas a significant difference is found in favour of castrates over intacts: *i.e.* 82% ± 3·0

FIG. 9.—The induction of cervico-vaginal carcinomas and papillomas by weekly applications of DMBA in intact and castrate rats given either methylthiouracil (M) or L-thyroxine (L) ± stilboestrol (S).

and 61% ± 4·0 thus reversing the proportion of induced sarcomas without additional medication, *i.e.* 25% ± 7·2 for castrates and 72% ± 6·85 for intacts. While in intacts the incidence of sarcomas is about the same with and without additional thyroactive compounds, that in castrates is greatly and significantly increased by the thyroactive compounds.

Since with DMBA alone the percentage of epithelial tumours induced is very low in castrates and intacts, it is not possible to demonstrate clearly a decrease due to additional medication. Thus only no significant change or increase in proportion are listed in Table V; for sarcomas no change, an increase as well as a decrease are given. The table indicates clearly the difference between intact and castrate rats and also the difference in response of the epithelial and the connective tissue to additional medication with thyroactive compounds. In intacts the incidence of epithelial tumours is increased by methylthiouracil given alone or combined with stilboestrol; there is no increase in sarcomas, but a decrease with the

TABLE V.—*Statistically Significant Changes in the Incidence of Epithelial and Sarcomatous Tumours due to Additional Treatments*

| Tumours | | Additional treatments | |
|---|---|---|---|
| Epithelial | Sarcomatous | Intacts | Castrates |
| N.S. | N.S. | L-thyroxine ± stilboestrol | — |
| N.S. | More | — | L-thyroxine ± stilboestrol |
| N.S. | Less | L-thyroxine plus methylthiouracil | — |
| More | N.S. | Methylthiouracil ± stilboestrol | L-thyroxine plus methylthiouracil |
| More | More | — | Methylthiouracil ± stilboestrol |

N.S. = Change not significant at 95% confidence level.

combined administration of methylthiouracil and L-thyroxine. In castrates more epithelial tumours occur after treatment with L-thyroxine plus methylthiouracil and with methylthiouracil alone or combined with stilboestrol; sarcomas occur in the same proportion with the combined thyroactive compounds or are promoted by either L-thyroxine $\pm$ stilboestrol or methylthiouracil $\pm$ stilboestrol.

### DISCUSSION

The effects of thyroactive compounds on the experimental induction of breast tumours are equivocal and if present inhibitory (Jull and Huggins, 1960; Helfenstein *et al.*, 1962; Newman and Moon, 1968) while they greatly accelerate and increase the induction of cervico-vaginal neoplasms particularly in castrates. To some extent this contrast might be attributed to the fact that in spayed rats tumours at either site occur only rarely (Shay, Harris and Gruenstein, 1952) and that the action of thyroactive compounds on the carcinogenesis of mammary tumours has been tested only in intact rats. Methylthiouracil, however, accelerates and increases the development of epithelial tumours at the cervix also in intact rats and this suggests that the difference at the two sites is real. Furthermore, the reduction in weight gain diminishes the incidence of breast tumours whether this is brought about by restriction of food intake, thyroidectomy (Gruenstein *et al.*, 1968; Jull and Huggins, 1960) or administration of propylthiouracil (Newman and Moon, 1968). The induction of cervico-vaginal tumours, however, is not affected by differences in the rate of gain in body weight (Table III) varying from 0·5% to 2·4% per week.

There is also no correlation between growth of the epithelium and stroma of the cervico-vaginal tract and carcinogenesis induced in them. Oestrogenic stimulation sufficient to promote growth in the stroma and epithelium of castrates, inhibits tumour formation in intacts and fails to promote it in castrates. The thyroactive compounds do not stimulate growth of these normal structures, but in castrates they increase the incidence of epithelial and sarcomatous tumours, and in intacts methylthiouracil increases that of papillomas and carcinomas. Stimulation of growth of the cervico-vaginal tissues by the addition of stilboestrol to the thyroactive medication tends to delay the formation of sarcomas (Fig. 5 and 6) in intacts and castrates, though there is a slight tendency to promote the appearance of epithelial tumours (Fig. 9). While in the oestrous cycle, for instance, growth of the stroma is coordinated with that of the epithelium this correlation is disturbed in carcinogenesis. Treatment by DMBA induces immediately some squamous hyperplasia in the atrophic castrate epithelium, but no thickening of the stroma is noticed before tumour formation. The preferential promotion by thyroactive substances of epithelial but not of sarcomatous tumours in intacts is further evidence of this disturbed relation.

The additional medication with thyroactive compounds produces more sarcomas faster in castrates than in intacts and this difference is significant with L-thyroxine $\pm$ stilboestrol treatment. Thus formation of connective tissue tumours in intacts is not maximal. Furthermore, in spayed animals both thyroactive compounds increase the incidence of sarcomas, but only methylthiouracil significantly affects that of epithelial tumours.

Thus the effects of thyroactive compounds on growth of the body or on that of the cervico-vaginal tract are not correlated with those on carcinogenesis. The additional medication may have systemic metabolic or immunological actions.

The incidence of carcinomas and of sarcomas in additionally treated castrates exceeds significantly that in intacts and there is a similar advantage in body weight which is partly accounted for by fat deposition. Without additional medication, however, castrates have fewer sarcomas than intacts and in rats treated with growth hormone a striking difference in weight gain between intacts and castrates is not paralleled by any significant difference in carcinogenesis (Table III). Of other metabolic changes analysed, neither diabetes nor insulin medication produces the same effects in intacts and castrates. A specific metabolic action of the various hormones cannot be exluded but it would have to account for a differential influence on epithelium and connective tissue as well as on intacts and spayed animals and for an only equivocal effect on carcinogenesis of the breast and vulva.

A possible immunological action has been found to be unlikely (Cherry and Glucksmann, 1960). While cortisone or whole body irradiation increases the tumour incidence in castrates, irradiation inhibits it in intacts presumably because of its sterilizing effect on the ovary. The effects of whole body exposure to X-rays and irradiation restricted to the pelvic region are identical in both intacts and castrates, though immunological competence is reduced much more by whole body than by partial body irradiation.

The oestrous cycle in rats shows a coordinated sequence of events in the epithelium and stroma of the uterus and of the cervico-vaginal tract and the changes are determined locally by the receptors in the tissue reacting to ovarian and other hormones which in their turn are regulated by the pituitary and hypothalamus. The topical administration of DMBA appears to interfere locally with the coordination of the epithelial and stromal components, *i.e.* with the activity or responsiveness of the receptors in these tissues and thus leads to a predominance of sarcomas over epithelial tumours in intact and pregnant animals (Glucksmann and Cherry, 1958) as well as in castrates. The cyclical activity of the pituitary and hypothalamus and with it of the ovary can be modified by the perinatal treatment of rats with oestradiol or with testosterone (Cherry and Glucksmann, 1968). If such animals are subjected to carcinogenic insults, the appearance of sarcomas as well as of epithelial tumours is delayed as compared with intact rats and with rats treated perinatally with either L-thyroxine or methylthiouracil (Fig. 7). The incidence of sarcomas in all the perinatally treated animals is of the same order and similar to that in castrate rats without additional medication. In oestrogenized mice and rats, however, more epithelial tumours than sarcomas are induced and they occur even without carcinogenic treatment (Dunn and Green, 1963; Kimura and Nandi, 1967; Takasugi and Bern, 1964; Cherry and Glucksmann, 1968), but not in rats treated perinatally with testosterone, methylthiouracil or L-thyroxine. In the latter group of animals the age-specific appearance of sarcomas resembles that in intact animals without additional treatment, while in oestrogenized rats it follows the pattern for castrates. These findings suggest that central factors may influence differentially the response to carcinogens of the stroma and epithelium of the target organ.

The central regulatory mechanism does not appear to be located primarily in the pituitary since there is no correlation between changes in the basophiles with the induction rate of either sarcomas or epithelial neoplasms. Thus in castrates given L-thyroxine concomitantly with DMBA castration cells are present in impressive numbers in the pituitary and there are more sarcomas than in similarly treated intacts. Without additional medication, however, a similar difference in the incidence of castration cells is associated with a significantly greater percentage of sarcomas in intact rats. The incidence of sarcomas and of epithelial tumours is the same in castrates given either methylthiouracil or L-thyroxine, though in the former but not in the latter group thyroidectomy cells are frequent. No very obvious changes in incidence of gonadotrophs and thyrotrophs appear in rats treated perinatally with oestradiol, testosterone, L-thyroxine or methylthiouracil though tumour incidence is modified. Some thyroidectomy cells are found in rats given methylthiouracil plus L-thyroxine and in intacts as well as in castrates the incidence of sarcomas is reduced significantly compared with animals given only one of the thyroactive compounds. On the other hand, epithelial neoplasms are significantly more frequent in castrates if the compounds are given simultaneously rather than separately but in intacts the difference is not significant.

Castration appears to affect the central regulatory mechanism and to make it more responsive to additional hormonal treatments as indicated by the greater effectiveness of additional medication in castrates than in intacts. Whether the inhibitory effect of continuous oestrogen administration on the induction of cervico-vaginal tumours in intact females (Glucksmann and Cherry, 1968) and on salivary gland tumours in intact male rats (Glucksmann and Cherry, 1966) is due to an effect on the receptor system of the target organs or on the central regulatory mechanisms cannot yet be decided. In the complex effects of additional hormonal treatments on carcinogenesis in various sites there may be an interplay of the target organs with the higher regulatory centres. Though responding to sex hormones the receptors in the cervico-vaginal tract, the vulva, the breast and salivary glands may respond differently to stimulation by other hormones and in their feedback to the higher centres. Thus thyroactive compounds have an only equivocal effect on the induction of breast cancers and on those of the vulva, a promoting action on those of the cervico-vaginal tract and inhibit carcinomas of the salivary gland in intact and castrate females (Glucksmann and Cherry, 1966).

### REFERENCES

BATHER, R. AND FRANKS, W. R.—(1952) *Cancer Res.*, **12**, 247.

CHERRY, C. P. AND GLUCKSMANN, A.—(1960) *Br. J. Cancer*, **14**, 489.—(1968) *Br. J. Cancer*, **22**, 728.

DUBNIK, C. S., MORRIS, H. P. AND DALTON, A. J.—(1950) *J. natn. Cancer Inst.*, **10**, 815.

DUNN, T. AND GREEN, A. W.—(1963) *J. natn. Cancer Inst.*, **31**, 425.

EAYRS, J. T. AND HOLMES, R. L.—(1964) *J. Endocr.*, **29**, 71.

GLUCKSMANN, A. AND CHERRY, C. P.—(1958) *Br. J. Cancer*, **12**, 32.—(1966) *Br. J. Cancer*, **20**, 760.—(1968) *Br. J. Cancer*, **22**, 545.—(1970) *Br. J. Cancer*, **24**, 333.

GRUENSTEIN, M., MERANZE, D. R., ACUFF, M. AND SHIMKIN, M. B.—(1968) *Cancer Res.*, **28**, 471.

HELFENSTEIN, J. E., YOUNG, S. AND CURRIE, A. R.—(1962) *Nature, Lond.*, **196**, 1108.

JULL, J. W. AND HUGGINS, C.—(1960) *Nature, Lond.*, **188**, 73.

KIMURA, T. AND NANDI, S.—(1967) *J. exp. Zool.*, **165**, 71.

NEWMAN, W. C. AND MOON, R. C.—(1968) *Cancer Res.*, **28**, 864.

SHAY, H., HARRIS, C. AND GRUENSTEIN, M.—(1952) *J. natn. Cancer Inst.*, **13**, 307.

SPENCER, J. G. C.—(1954) *Br. J. Cancer*, **8**, 393.

TAKASUGI, N. AND BERN, H. A.—(1964) *J. natn. Cancer Inst.*, **33**, 855.

WILKINS, R. H. AND MORTON, D. L.—(1963) *Cancer, N.Y.*, **16**, 558.

WILLIAMS, M. W. AND WILLIAMS, C. S.—(1965) *Cancer Chemother. Rep.*, **45**, 1.

VAZQUEZ-LOPEZ, E.—(1949) *Br. J. Cancer*, **3**, 401.


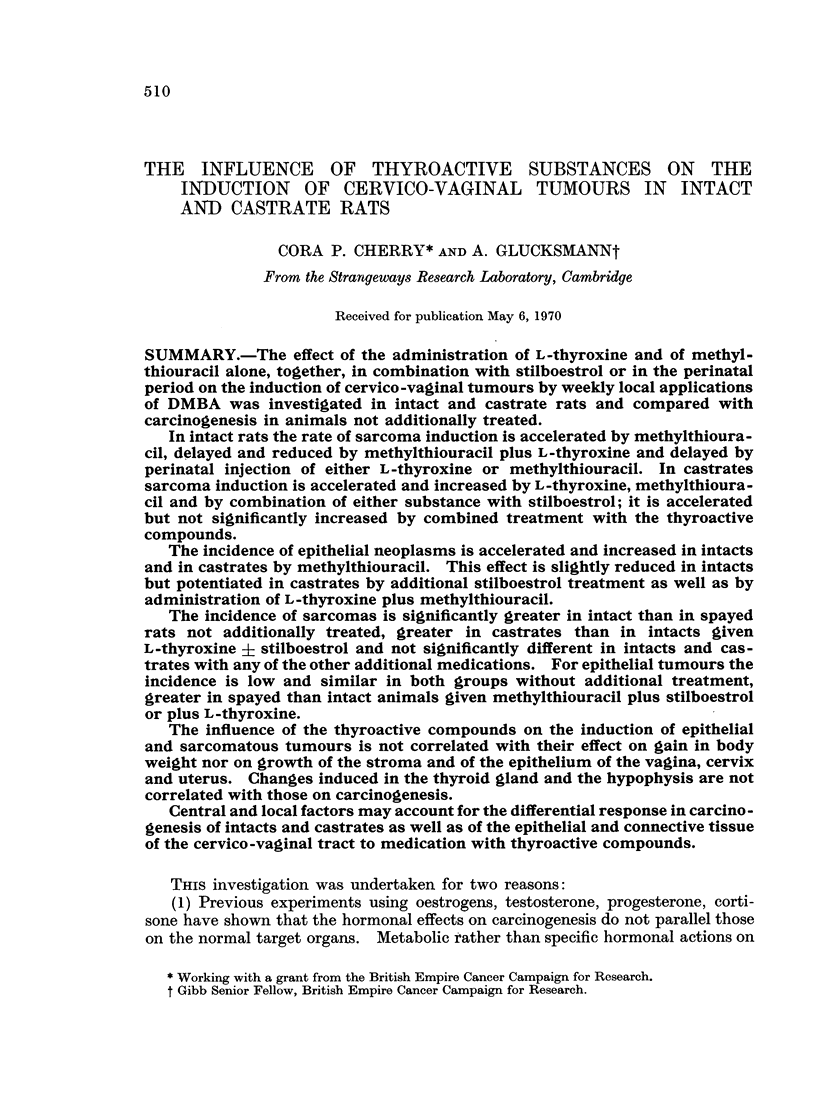

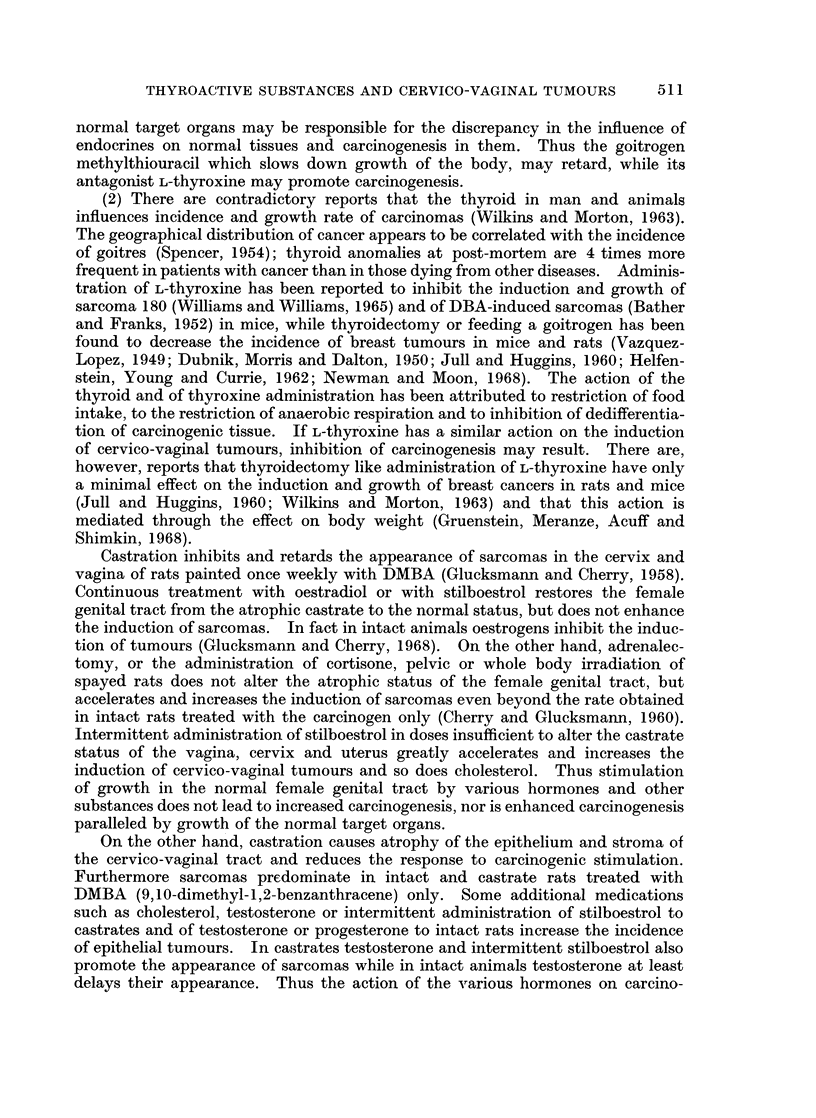

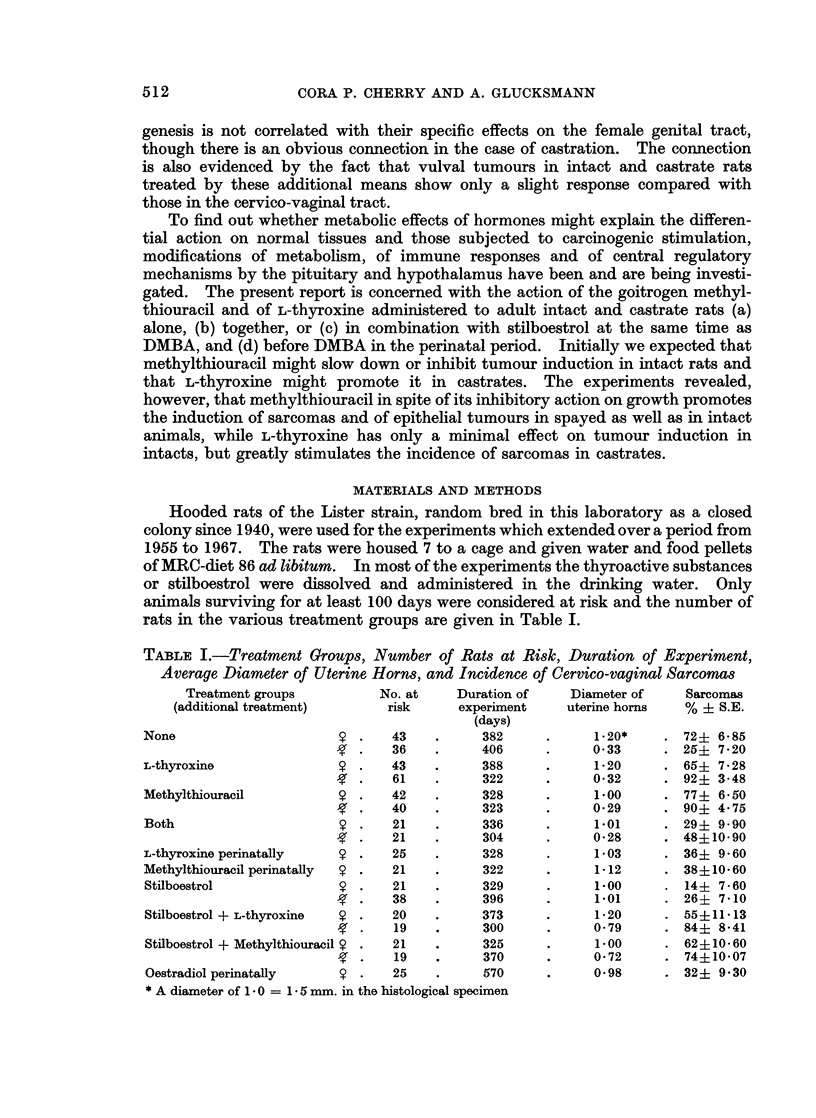

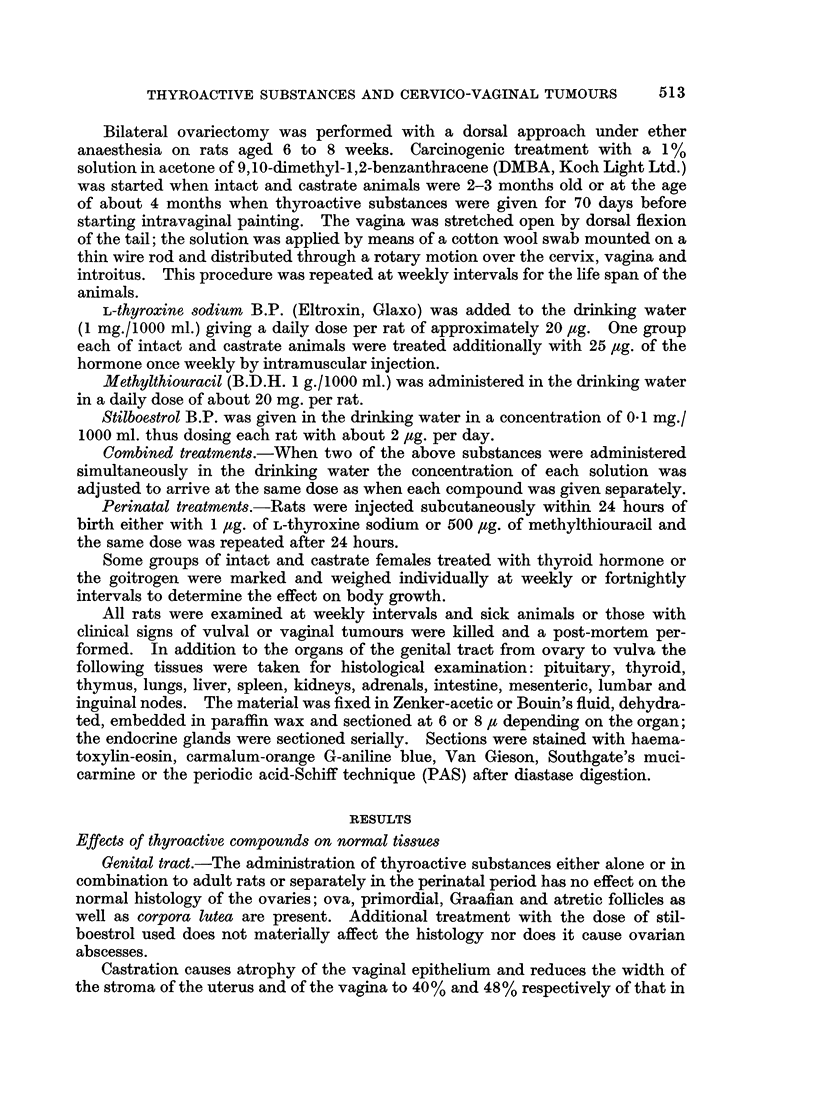

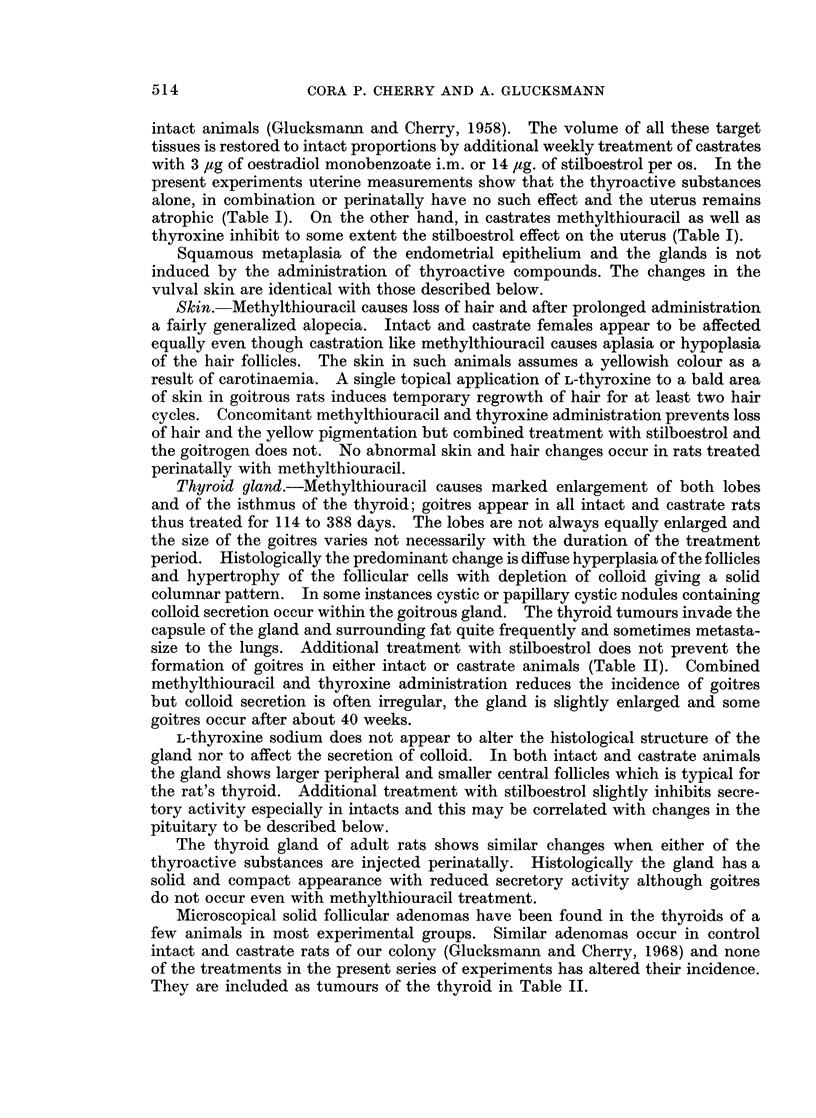

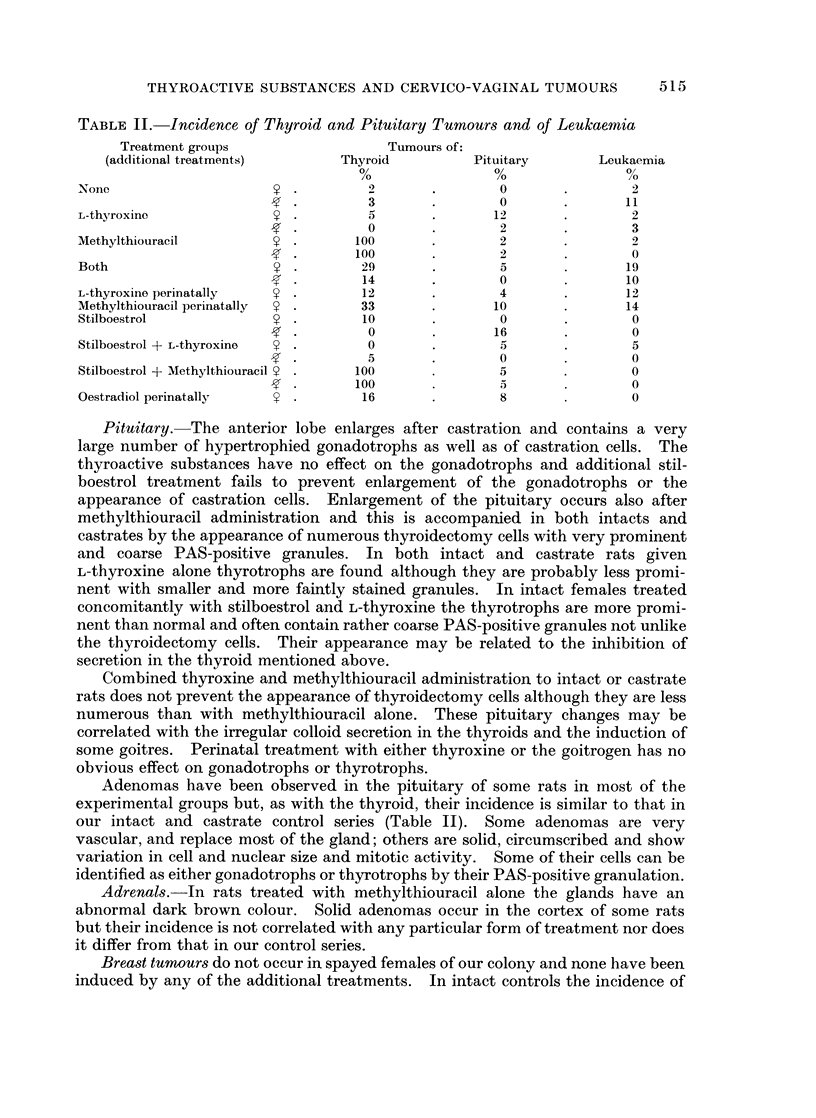

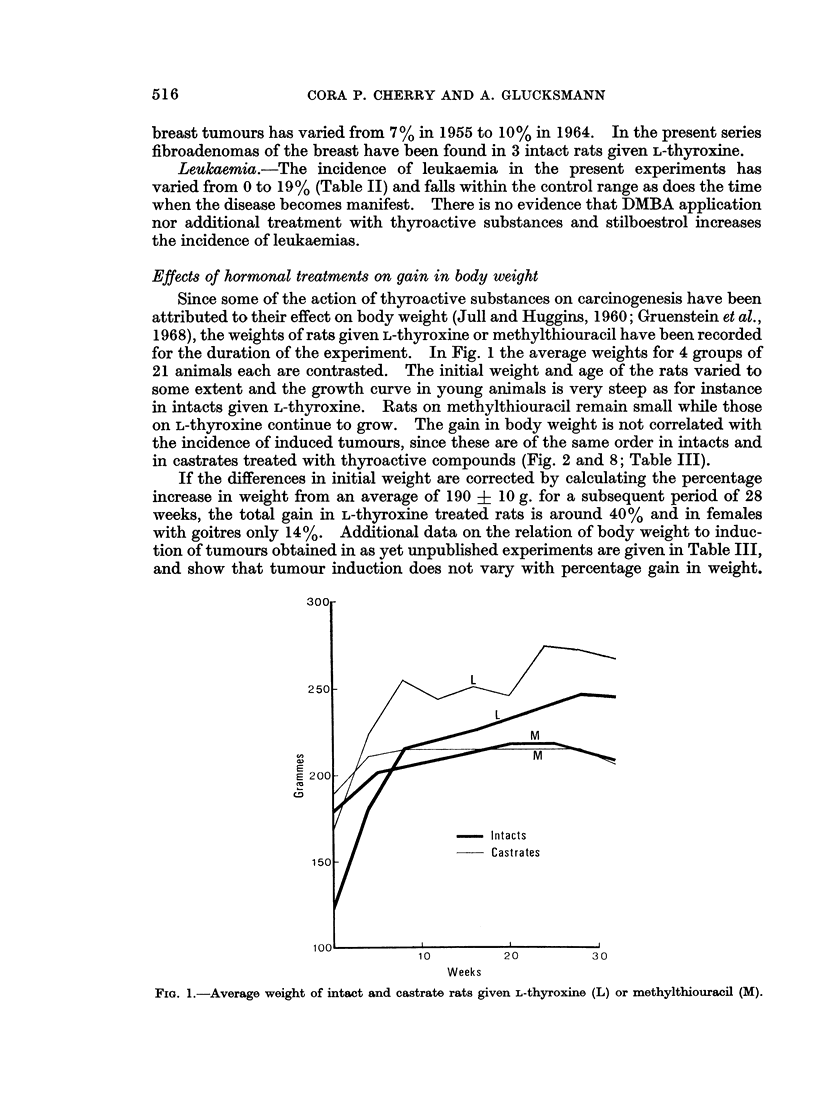

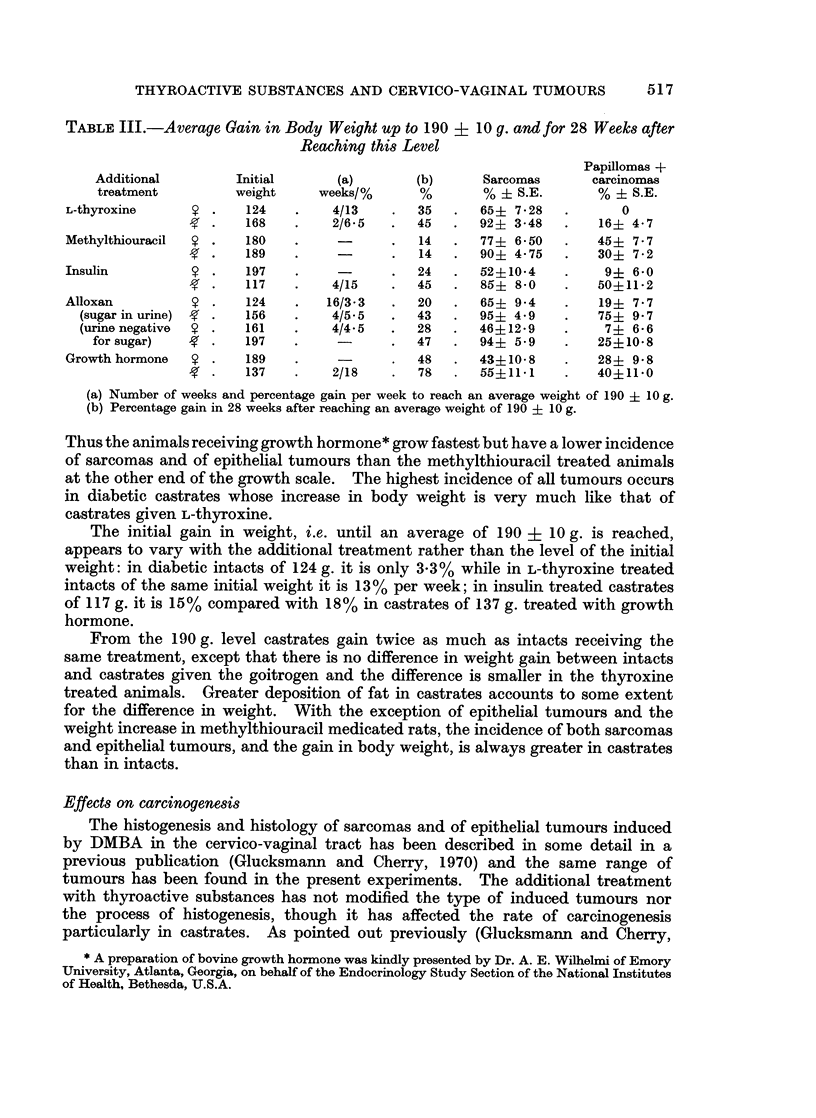

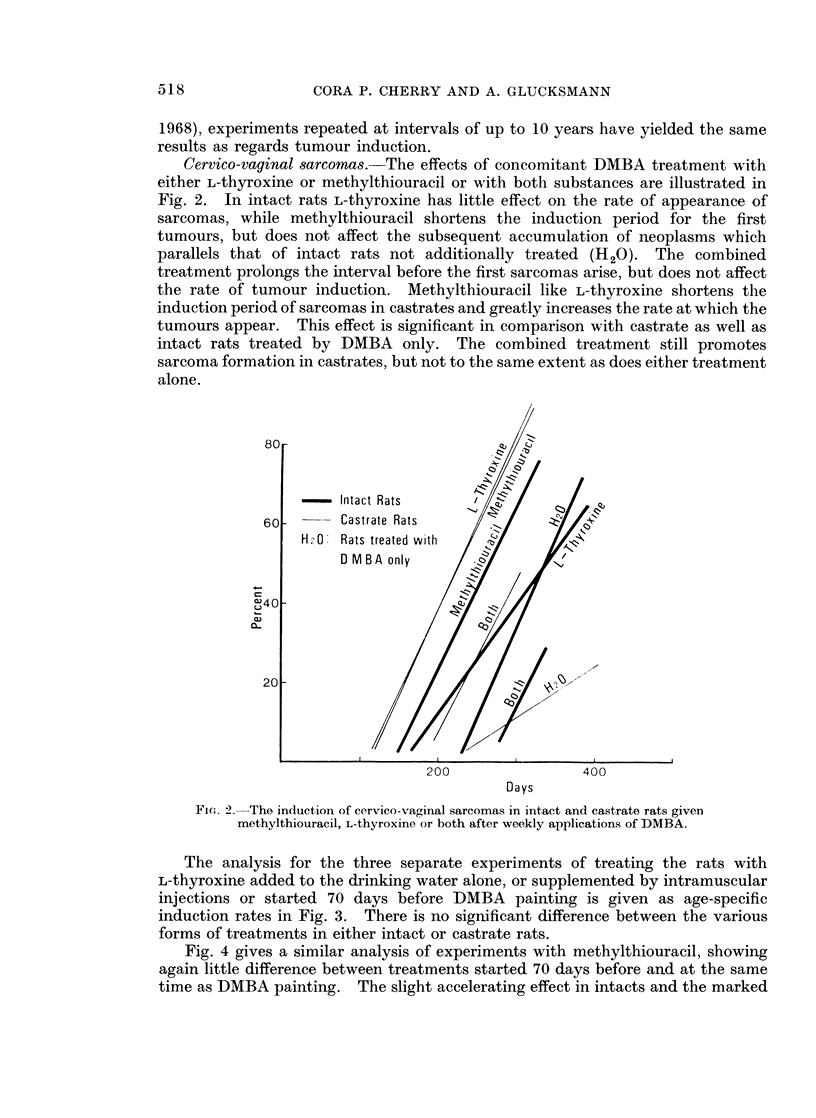

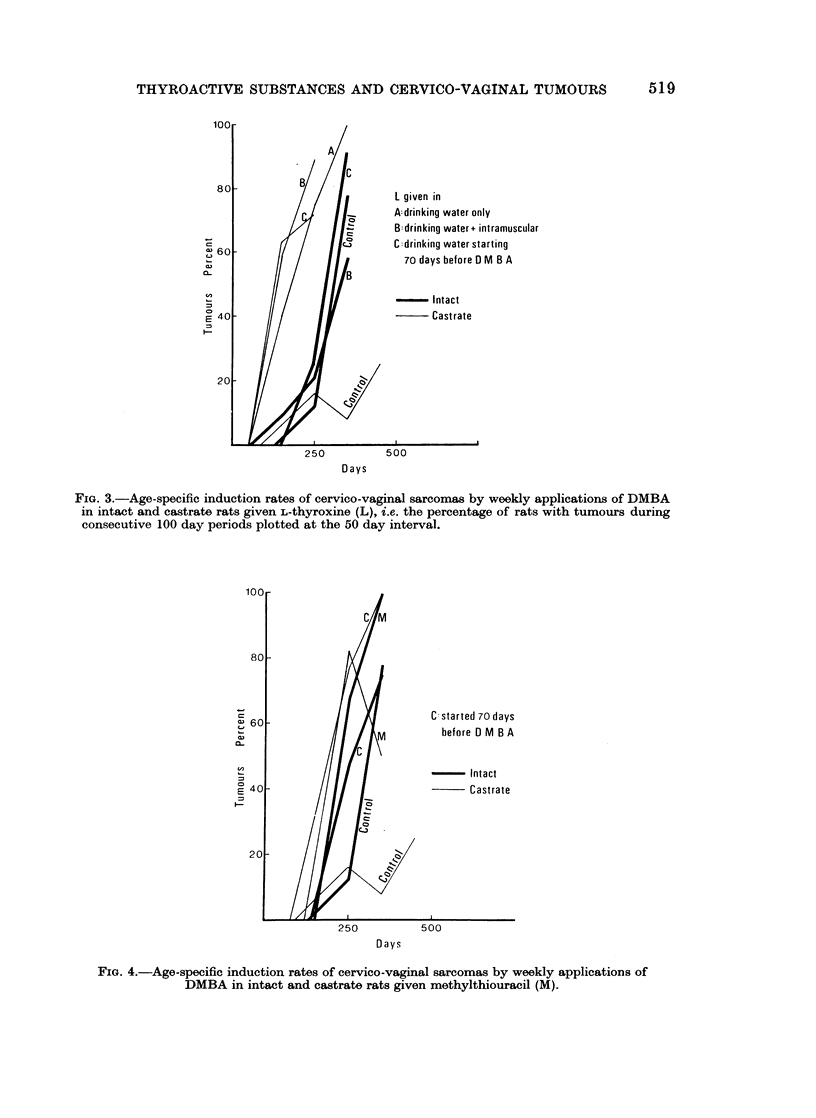

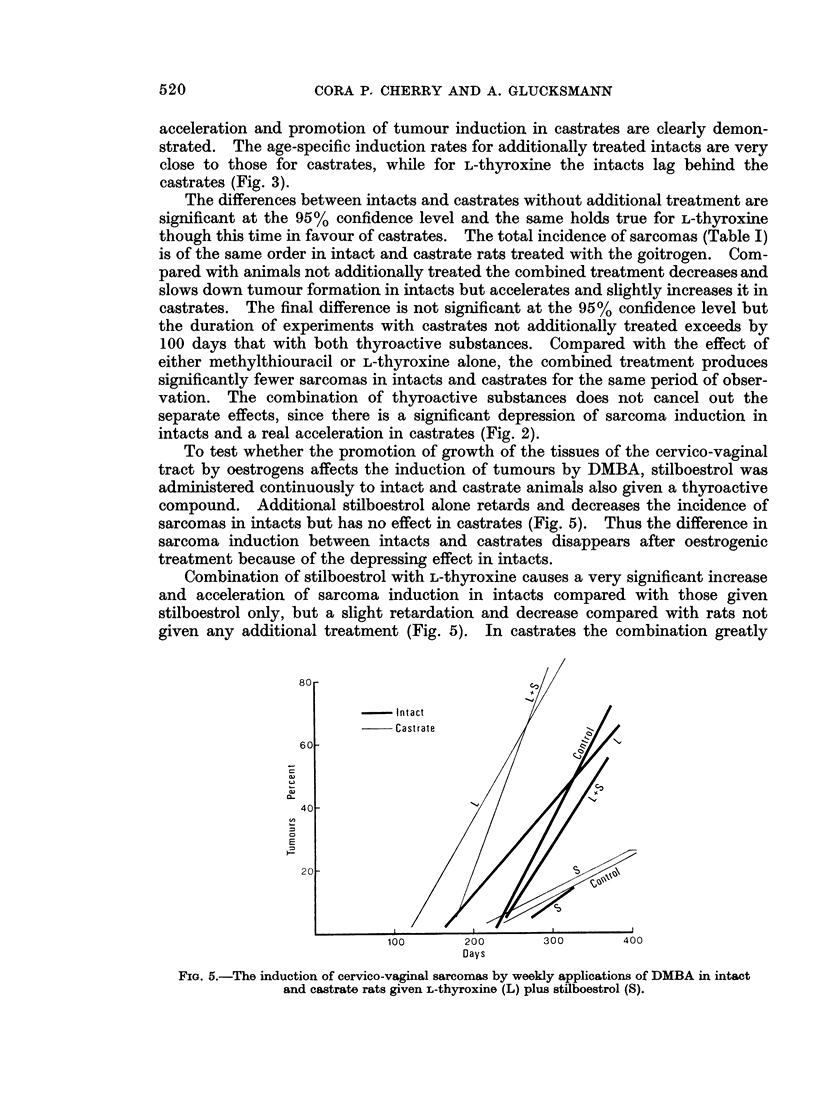

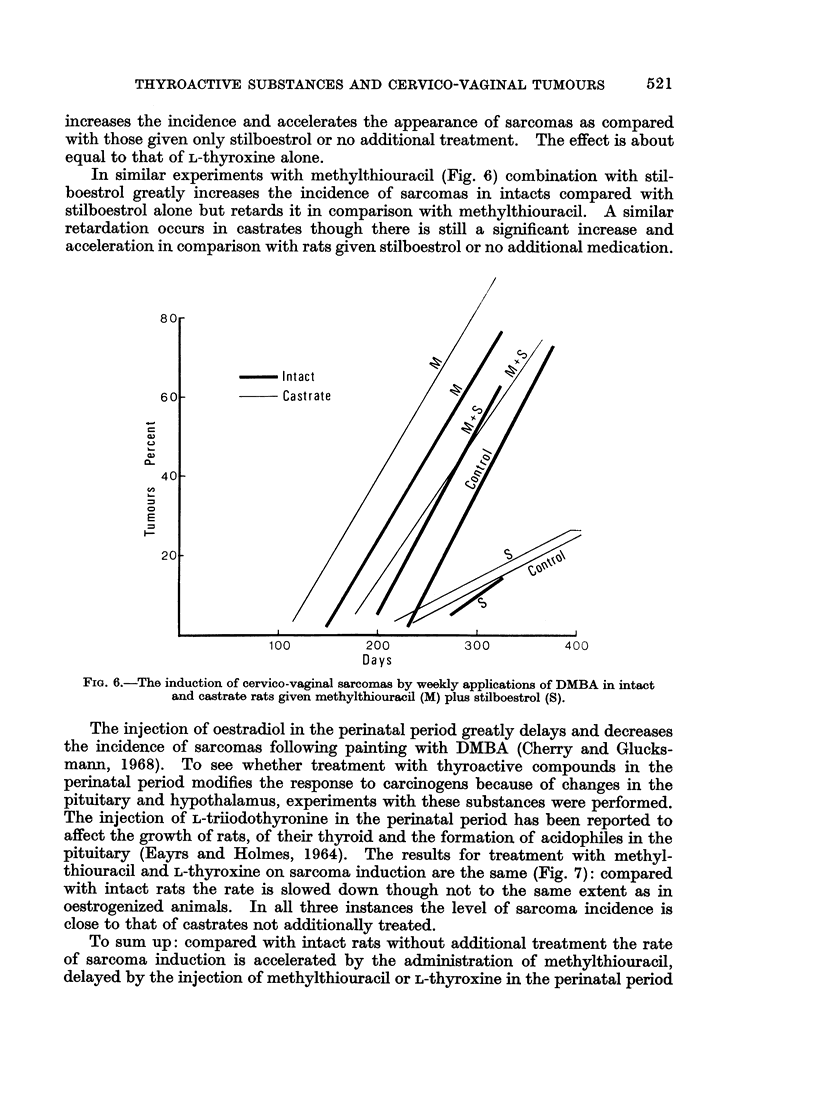

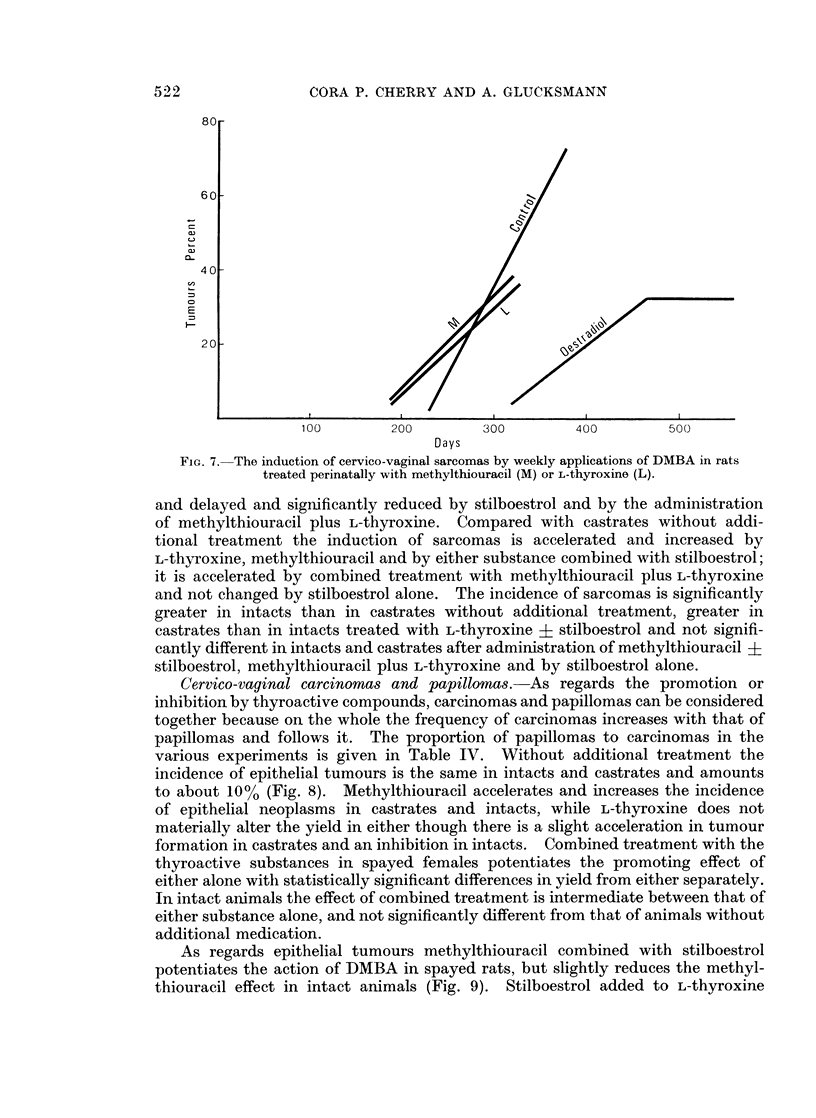

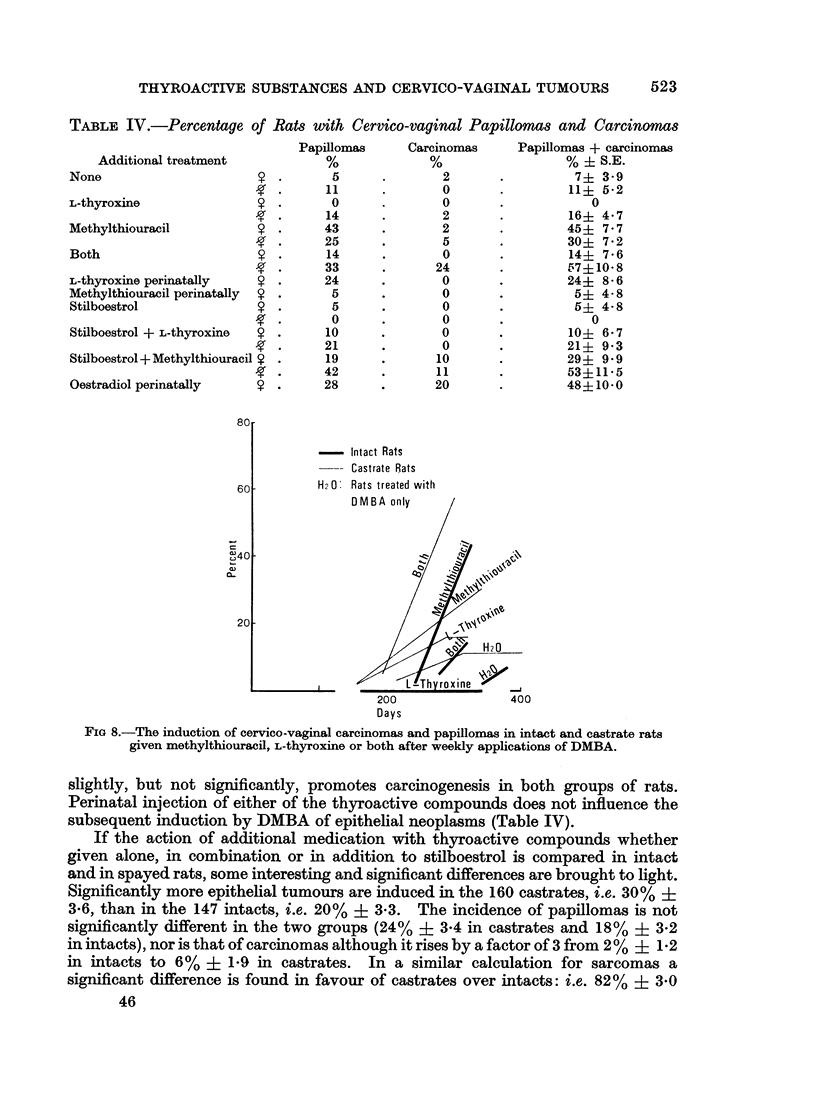

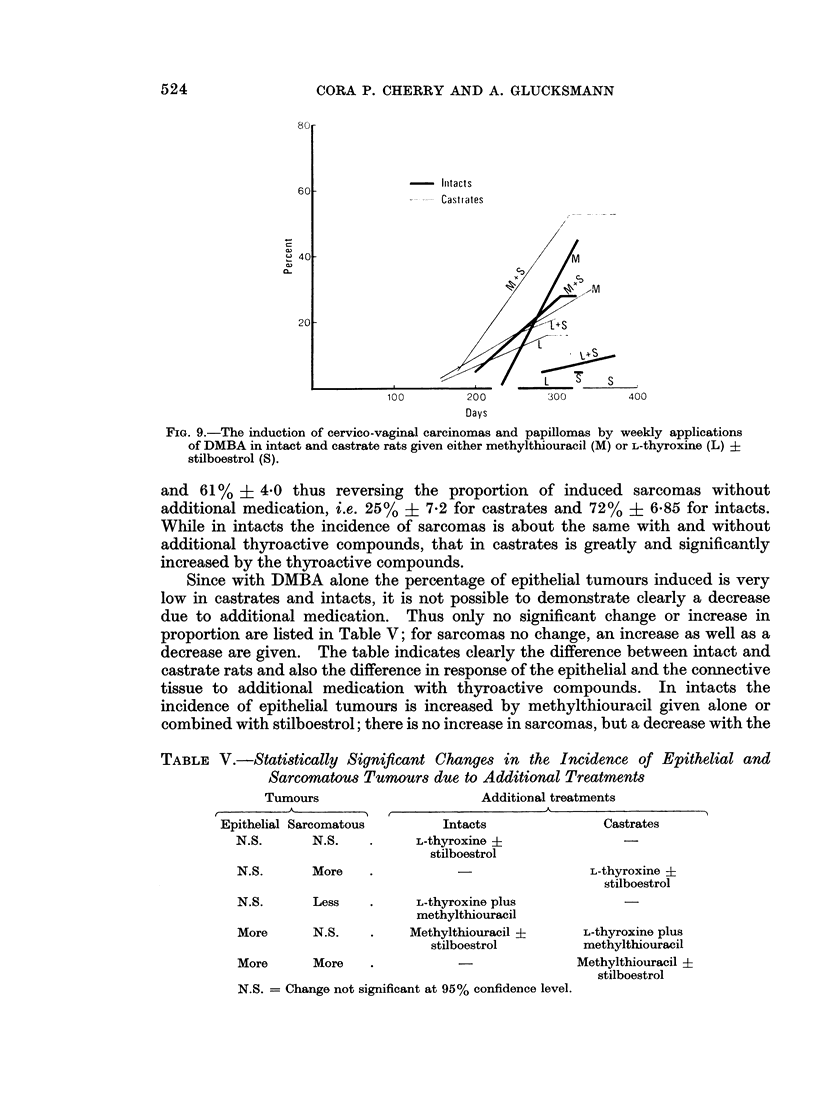

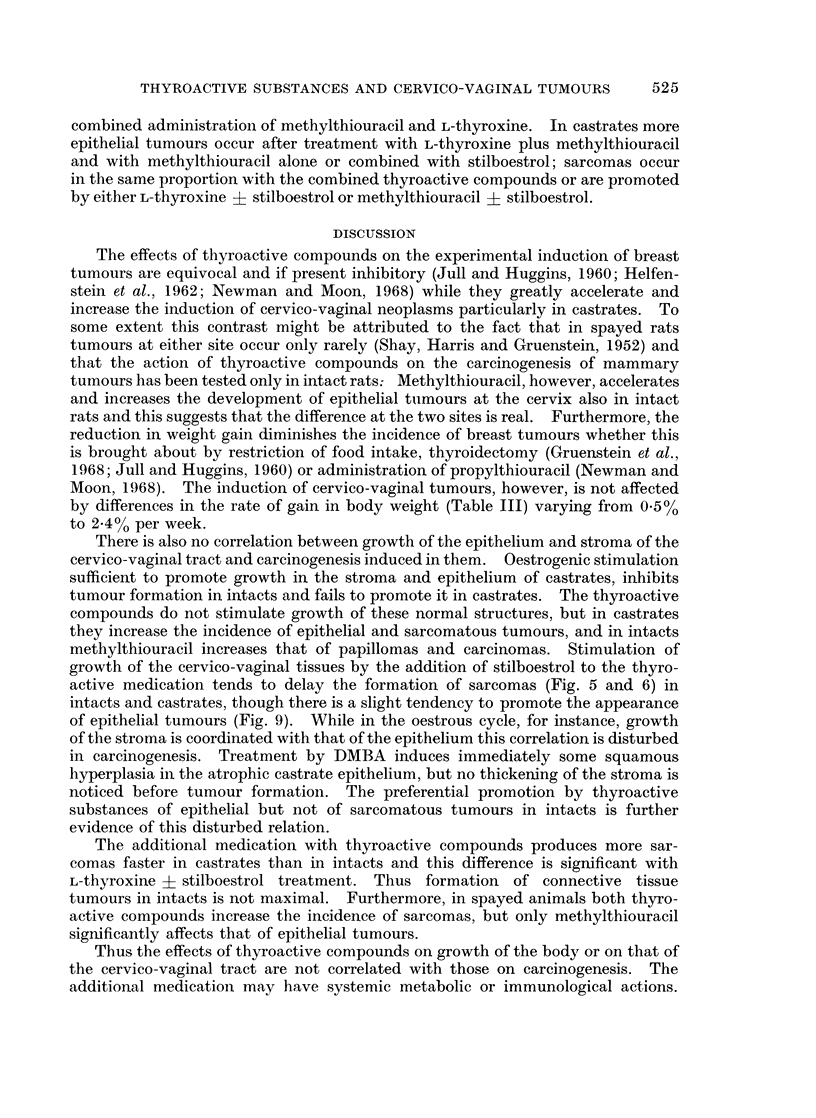

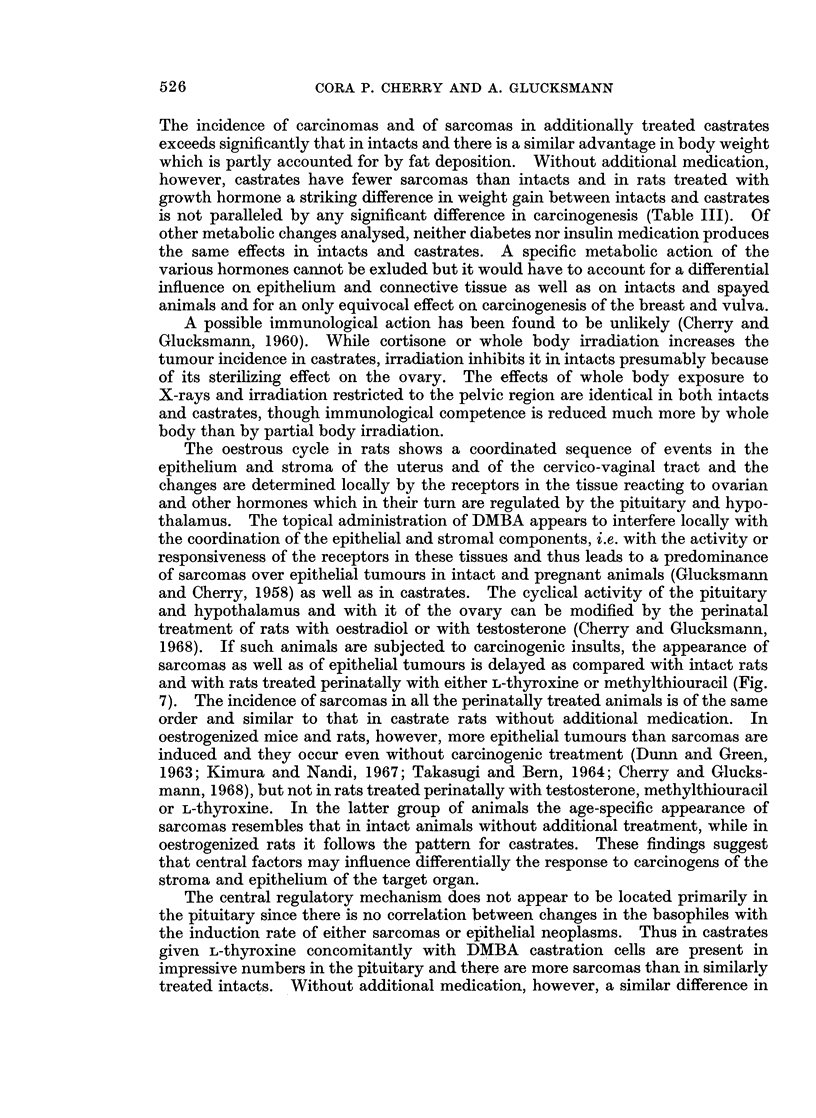

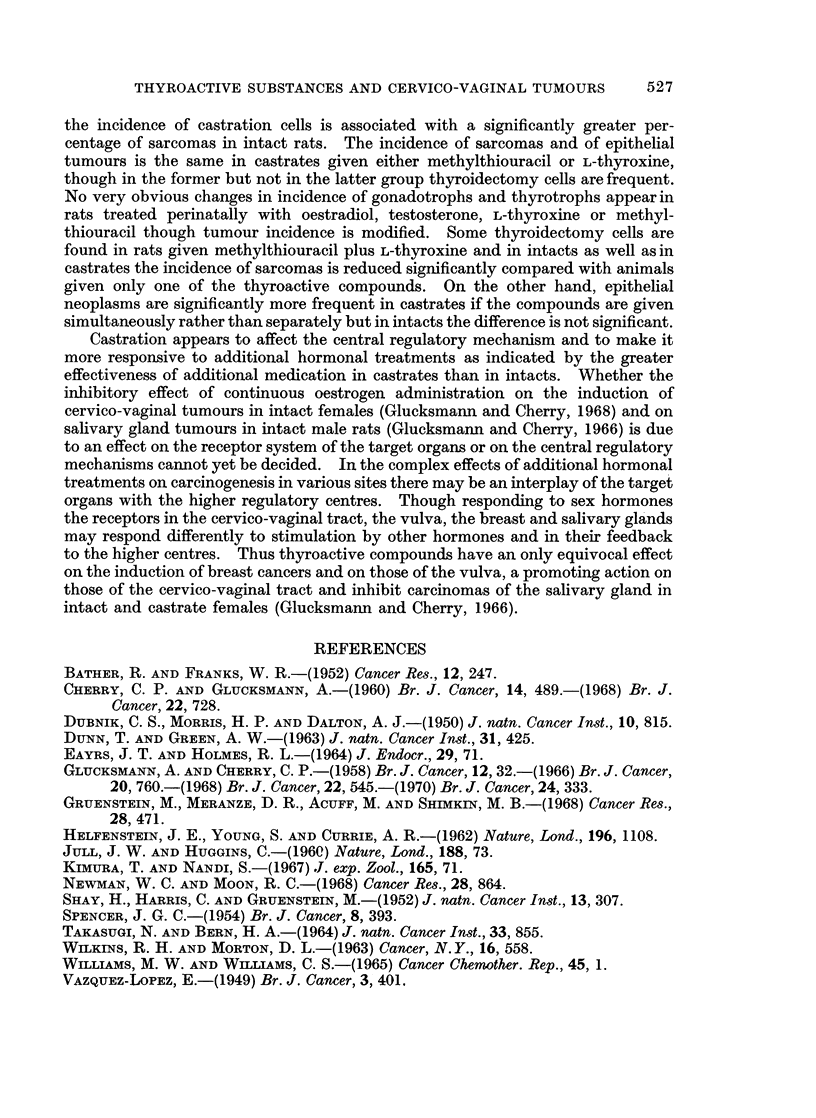


## References

[OCR_01154] Cherry C. P., Glucksmann A. (1968). The induction of cervico-vaginal tumours in oestrogenised and androgenised rats.. Br J Cancer.

[OCR_01158] DUBNIK C. S., MORRIS H. P., DALTON A. J. (1950). Inhibition of mammary-gland development and mammary-tumor formation in female C3H mice following ingestion of thiouracil.. J Natl Cancer Inst.

[OCR_01160] DUNN T. B., GREEN A. W. (1963). CYSTS OF THE EPIDIDYMIS, CANCER OF THE CERVIX, GRANULAR CELL MYOBLASTOMA, AND OTHER LESIONS AFTER ESTROGEN INJECTION IN NEWBORN MICE.. J Natl Cancer Inst.

[OCR_01164] Glucksmann A., Cherry C. P. (1968). The effect of oestrogens, testosterone and progesterone on the induction of cervico-vaginal tumours in intact and castrate rats.. Br J Cancer.

[OCR_01166] Gruenstein M., Meranze D. R., Acuff M., Shimkin M. B. (1968). The role of the thyroid in hydrocarbon-induced mammary carcinogenesis in rats.. Cancer Res.

[OCR_01170] HELFENSTEIN J. E., YOUNG S., CURRIE A. R. (1962). Effect of thiouracil on the development of mammary tumours in rats induced with 9,10-dimethyl-1,2-benzanthracene.. Nature.

[OCR_01174] Newman W. C., Moon R. C. (1968). Chemically induced mammary cancer in rats with altered thyroid function.. Cancer Res.

[OCR_01176] SHAY H., HARRIS C., GRUENSTEIN M. (1952). Influence of sex hormones on the incidence and form of tumors produced in male or female rats by gastric instillation of methylcholanthrene.. J Natl Cancer Inst.

[OCR_01179] TAKASUGI N., BERN H. A. (1964). TISSUE CHANGES IN MICE WITH PERSISTENT VAGINAL CORNIFICATION INDUCED BY EARLY POSTNATAL TREATMENT WITH ESTROGEN.. J Natl Cancer Inst.

[OCR_01183] VAZQUEZ-LOPEZ E. (1949). The effects of thiourea on the development of spontaneous tumours on mice.. Br J Cancer.

[OCR_01180] WILKINS R. H., MORTON D. L. (1963). The influence of thyroid hormone analogues on an isotransplanted spontaneous mammary adenocarcinoma in mice.. Cancer.

